# Supplementary material for: Electronic Polarizability Tunes the Function of the Human Bestrophin 1 Cl− Channel
Source: bioRxiv. 2024 Aug 8:2023.11.14.567055. Originally published 2023 Nov 14. Preprint. [Version 2] doi: 10.1101/2023.11.14.567055 (PMC10680768; doi:10.1101/2023.11.14.567055)
Supplement: 1 [file NIHPP2023.11.14.567055V2-supplement-1.pdf]

bioRxiv preprint doi: https://doi.org/10.1101/2023.11.14.567055; this version posted August 8, 2024. The copyright holder for this preprint (which was not certified by peer review) is the author/funder, who has granted bioRxiv a license to display the preprint in perpetuity. It is made available under aCC-BY 4.0 International license.

Supplementary Methodological Details

Full protein embedded simulations

The full protein systems were prepared using a multiscale procedure. The protein is first coarse-grained (CG) and then embedded into a POPC bilayer and solvating with water and ~ 0.5 M NaCl using Martini version 2.2 (1) and GROMACS 2021 package (2). This system is subject to 100 ns of equilibration in CG before being converted to atomistic representation with the CG2AT protocol (3). An equilibration period of 20 ns followed by a production run of 100 ns using the c36m forcefield (4) and mTIP3P water model was performed. The temperature was maintained at 310 K with coupling constant 1.0 ps by the Nosé-Hoover thermostat (5). Pressure was maintained at 1 bar with coupling constant 5.0 ps by the Parrinello-Rahman barostat (6). Short-range electrostatics were treated with the Verlet cutoff scheme at 1.2 nm cutoff and long-range electrostatics were treated with PME(7). C-alpha atoms were placed under harmonic restraints with a force constant of 1000 kJ/mol/nm^2 to prevent the structure from deviating too much from the experimental structure.

bioRxiv preprint doi: https://doi.org/10.1101/2023.11.14.567055; this version posted August 8, 2024. The copyright holder for this preprint (which was not certified by peer review) is the author/funder, who has granted bioRxiv a license to display the preprint in perpetuity. It is made available under aCC-BY 4.0 International license.

Figure S1

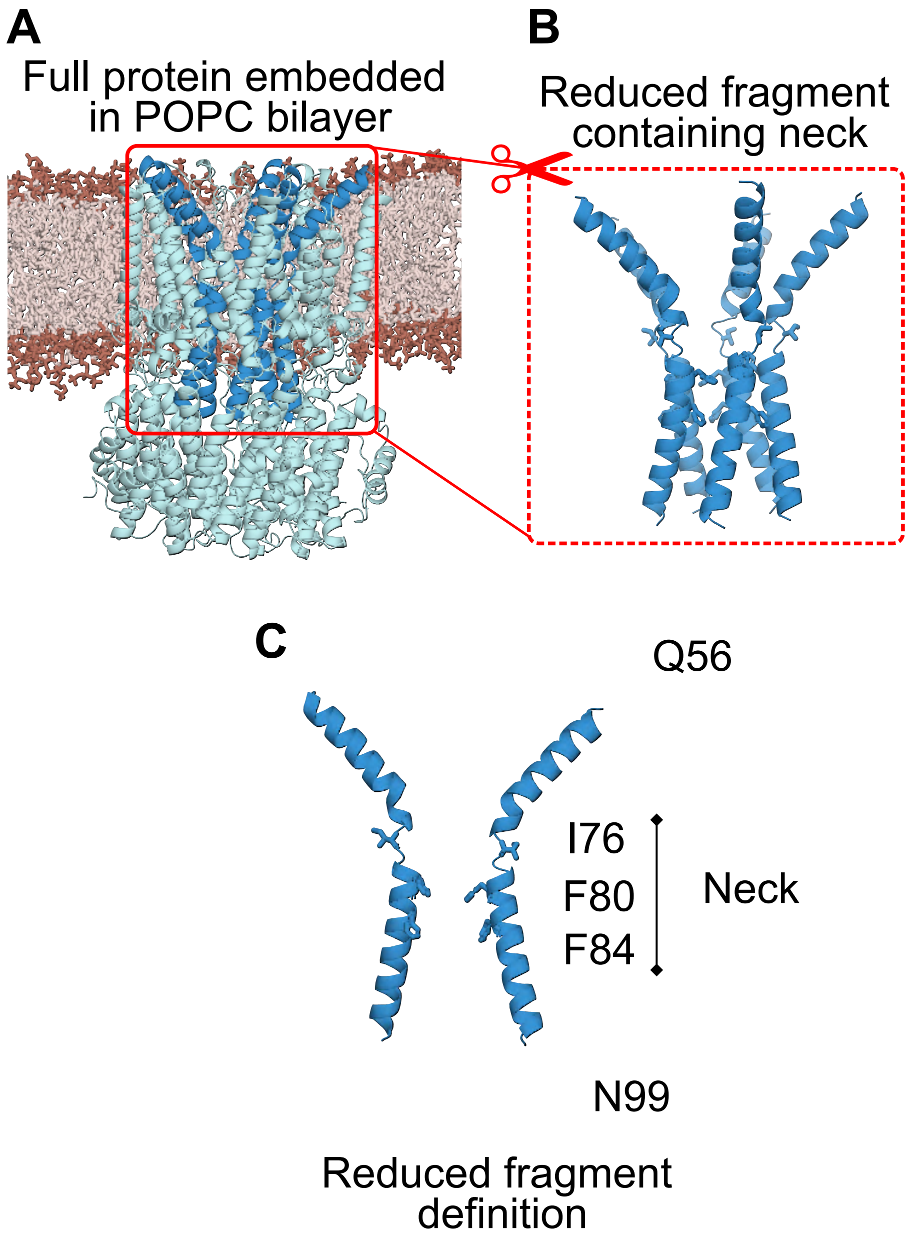

***Figure S1**:* **A** Full protein (cyan) embedded in POPC bilayer (brown). **B** Protein fragment system used in simulations due to the high computational demands of the AMOEBA forcefield. The full protein embedded system in solution contains ~200500 atoms. **C** The protein was truncated at Q56 and N99 such that the remaining fragment system contains the conserved hydrophobic neck region of interest (I76, F80, F84). The reduced protein fragment in solution contains ~57000 atoms. Water and ions are omitted for clarity.

bioRxiv preprint doi: https://doi.org/10.1101/2023.11.14.567055; this version posted August 8, 2024. The copyright holder for this preprint (which was not certified by peer review) is the author/funder, who has granted bioRxiv a license to display the preprint in perpetuity. It is made available under aCC-BY 4.0 International license.

Figure S2

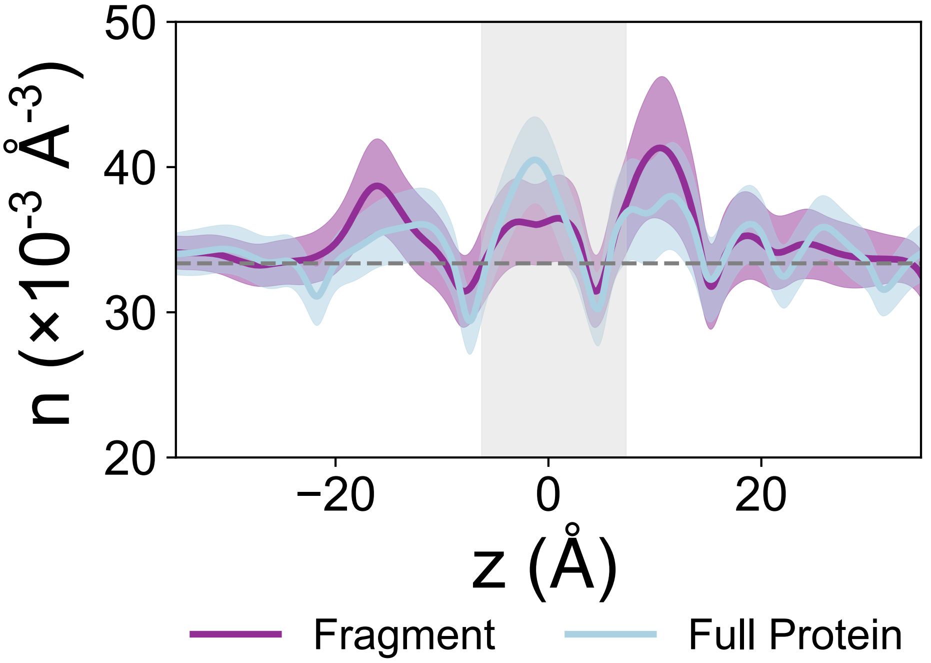

Figure S2: Protein fragment system validation. Time-averaged water density profiles within the pore for the full protein embedded system (light blue) compared with the protein fragment in solution (purple) using c36m. The shaded region represents the neck region, and the dashed grey line corresponds to the density of bulk water (33.37 nm^{-3}). Confidence bands represent the standard deviation over the simulation.

bioRxiv preprint doi: https://doi.org/10.1101/2023.11.14.567055; this version posted August 8, 2024. The copyright holder for this preprint (which was not certified by peer review) is the author/funder, who has granted bioRxiv a license to display the preprint in perpetuity. It is made available under aCC-BY 4.0 International license.

Figure S3

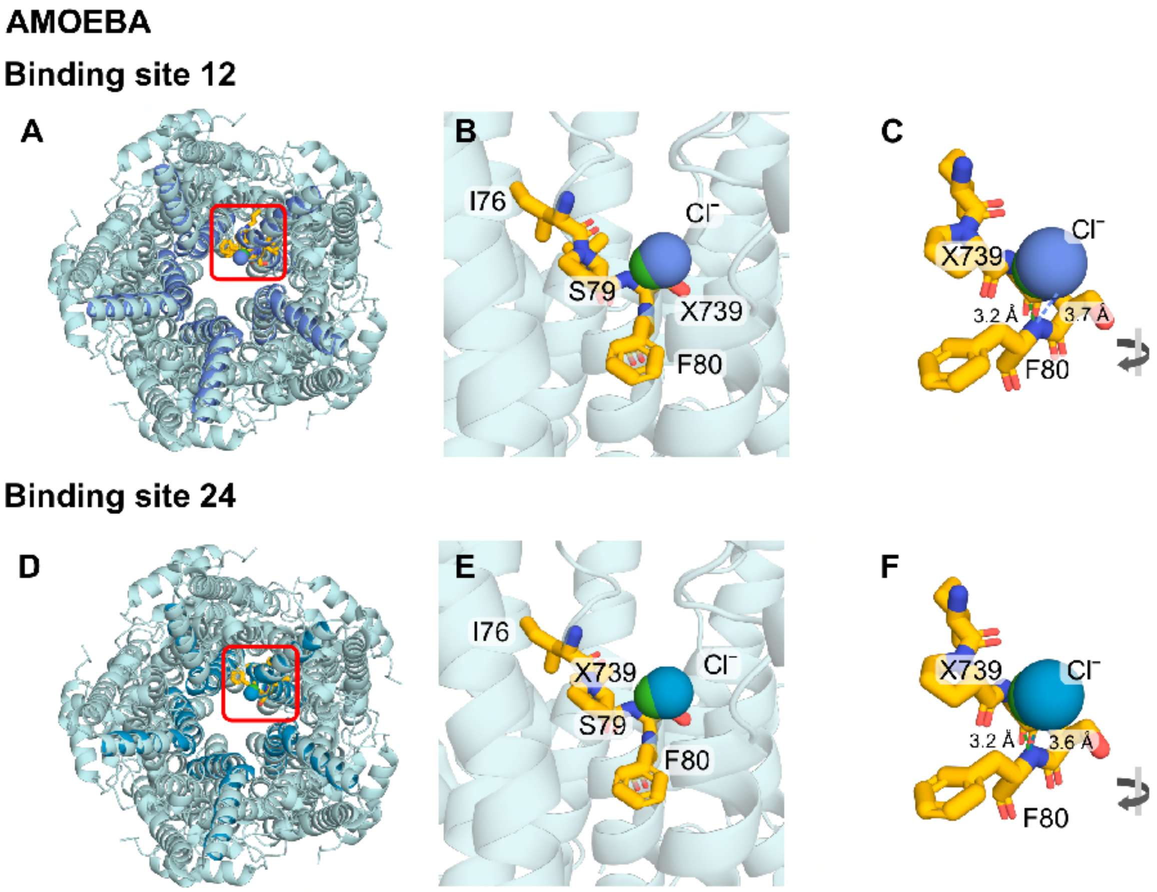

G

Binding Site	Site Residence Time (ns)	k_{off}	Duration (ns)	Occupancy (%)	R-squared	Distance to N of F80 (Å)	Distance to X739 in PDB (Å)
12	0.29	3.5	0.39	30	0.9995	3.7	0.7
15	0.59	1.7	0.37	36	0.9994	3.5	0.4
24	0.25	4.1	0.34	38	0.9978	3.6	0.7

Figure S3: Alternative AMOEBA binding sites of the open state structure (PDB ID 8D1O). **A-C** Binding site 12 and **D-F** show binding site 24. Table **G** gives the binding site statistics where binding site 15 is the site shown in Figure 2 in the main manuscript.

bioRxiv preprint doi: https://doi.org/10.1101/2023.11.14.567055; this version posted August 8, 2024. The copyright holder for this preprint (which was not certified by peer review) is the author/funder, who has granted bioRxiv a license to display the preprint in perpetuity. It is made available under aCC-BY 4.0 International license.

Figure S4

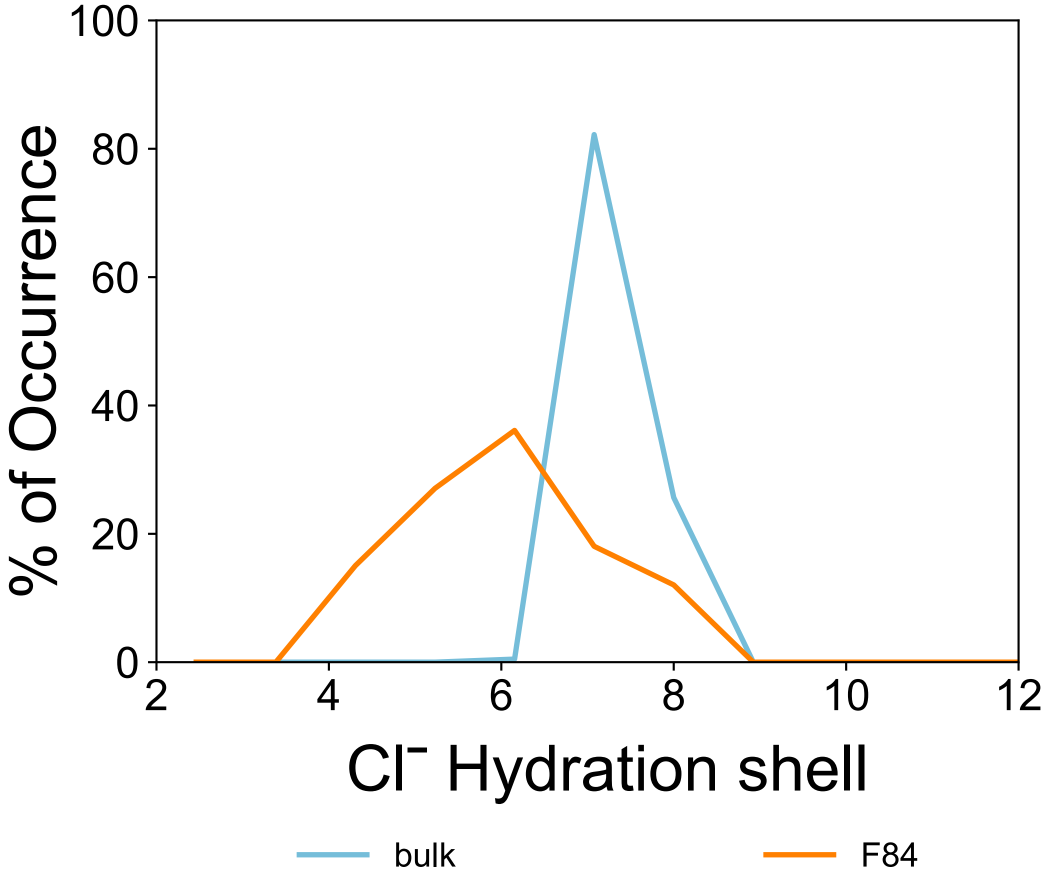

Figure S4: First hydration shell number of chloride ions in the partially open state structure (PDB ID 8D1K) in bulk (blue) compared with at the z-position corresponding to F84 (orange) within the pore in the AMOEBA simulation. Chloride loses 1-2 water molecules in its first hydration shell at F84 relative to bulk. This is comparable to the dehydration that occurs at F80 in the fully open state (PDB ID 8D1O).

bioRxiv preprint doi: https://doi.org/10.1101/2023.11.14.567055; this version posted August 8, 2024. The copyright holder for this preprint (which was not certified by peer review) is the author/funder, who has granted bioRxiv a license to display the preprint in perpetuity. It is made available under aCC-BY 4.0 International license.

Supporting information references

1. D. H. De Jong, *et al.*, Improved parameters for the martini coarse-grained protein force field. *J Chem Theory Comput* **9**, 687–697 (2013).
2. M. J. Abraham, *et al.*, Gromacs: High performance molecular simulations through multi-level parallelism from laptops to supercomputers. *SoftwareX* **1–2**, 19–25 (2015).
3. O. N. Vickery, P. J. Stansfeld, CG2AT2: An Enhanced Fragment-Based Approach for Serial Multi-scale Molecular Dynamics Simulations. *J Chem Theory Comput* **17**, 6472–6482 (2021).
4. J. Huang, *et al.*, CHARMM36m: An improved force field for folded and intrinsically disordered proteins. *Nat Methods* **14**, 71–73 (2016).
5. D. J. Evans, B. L. Holian, The Nose-Hoover thermostat. *J Chem Phys* **83**, 4069–4074 (1985).
6. M. Parrinello, A. Rahman, Polymorphic transitions in single crystals: A new molecular dynamics method. *J Appl Phys* **52**, 7182–7190 (1981).
7. U. Essmann, *et al.*, A smooth particle mesh Ewald method. *J Chem Phys* **103**, 8577–8593 (1995).
